# Robust Adaptive Lasso method for parameter’s estimation and variable selection in high-dimensional sparse models

**DOI:** 10.1371/journal.pone.0183518

**Published:** 2017-08-28

**Authors:** Abdul Wahid, Dost Muhammad Khan, Ijaz Hussain

**Affiliations:** 1 Department of Statistics, Abdul Wali Khan University Mardan, Khyber Pakhtunkhwa, Pakistan; 2 Department of Statistics, Quiad-i-Azam University Islamabad, Islamabad, Pakistan; National University of Defense Technology, CHINA

## Abstract

High dimensional data are commonly encountered in various scientific fields and pose great challenges to modern statistical analysis. To address this issue different penalized regression procedures have been introduced in the litrature, but these methods cannot cope with the problem of outliers and leverage points in the heavy tailed high dimensional data. For this purppose, a new Robust Adaptive Lasso (RAL) method is proposed which is based on pearson residuals weighting scheme. The weight function determines the compatibility of each observations and downweight it if they are inconsistent with the assumed model. It is observed that RAL estimator can correctly select the covariates with non-zero coefficients and can estimate parameters, simultaneously, not only in the presence of influential observations, but also in the presence of high multicolliearity. We also discuss the model selection oracle property and the asymptotic normality of the RAL. Simulations findings and real data examples also demonstrate the better performance of the proposed penalized regression approach.

## 1 Introduction

Variable selection plays a vital role in modern statistical modeling and machine learning, especially for models in which a large number of predictors and minimum observations, known as high dimensionality. Introducing many predictors in the regression models will result in reducing bias in model, but we wish to select a parsimonious set of important covariates for the efficient prediction. Therefore, variable selection is, then, essential to identify important variables, and produces more interpretable models with better prediction power.

The penalization methods are very useful in this field, and there are a large body of literature on this problems recently. The least absolute shrinkage and selector operator (LASSO), which was introduced by [1], is one of the key steps in coefficient estimation and predictor selection simultaneously, and was further studied by [2] and [3]. [4] proposed bridge regression that depends on *L*_*q*_ penalty. [5] proposed correlation based penalty to encourage a grouping effect, and also does well when there is high correlation among explanatory variables. It does not do as well when the correlation is perfect [6]. The smoothly clipped absolute deviation (SCAD) penalty, proposed by [7], enjoys the oracle property, but this criterion is non-convex, which is a drawback since it makes the computation much more difficult. [8] proposed the adaptive LASSO which is convex and reduces the possible bias. Therefore, it consistently selects the model. He also proved the oracle property of proposed method. Moreover, several other penalized methods had been proposed, such as [9, 10].

Recently, many sparse learning and classification algorithms have been proposed. [11] proposed weighted sparse representation based classification (WSRC) method which is direct extention of SRC, integrates the locality to sparse coding. WSRC provides good results in lower dimensional subspaces. [12], developed a new classification algorithm called Representative Vector Machines (RVM’s). The comprehensive experimental evaluation demonstrate the effectiveness of proposed method over other classifiers. [13] also presents a comprehensive survey of various aspects of structured sparsity-inducing feature selection (SSFC) methods on feature selection. [14] and [15] have proposed a new subspase learning algorithm called discriminant sparse neihborhood preserving embedding(DSNPE) and a robust feature extraction method for face recognition based on least squares regression, respectively.

Robust penalized techniques, such as the least absolute deviation and quantile regression, have been used for predictor selection in the case of fixed dimensionality, for instance, [16, 17, 18, 19]. [17] studied the *L*_1_-penalized LAD regression. The penalized composite likelihood method was proposed in [20] for robust estimation in high-dimensions with focus on the efficiency of the method. [21, 22] studied the *L*_1_-penalized quantile regression in high-dimensional sparse models.

In this paper, we develop a robust penalized method for estimating regression coefficients and predictor selection. We consider a weighted likelihood estimating equation with *L*_1_ penalty of [8] which employs a re-weighting of the components of the likelihood score function. The proposed approach is useful when the model is in doubt or when outliers are present in the data. This work is based on a recent proposal by [23]. Their work requires to attaching weights to score function values of each observation in such a way that the weights are close to one when the residuals describe the match between the empirical and the true model distribution functions. On the other hand, for large residuals there is a mismatch, and the corresponding likelihood function may require downweighting in order to obtain a robust solution.

However, the [24] and [25] articles remain the pioneers in this particular area of research. According to [24], this approach of downweighting points that are large residuals outliers and as well as both residuals outliers and high leverage points.

In the present work, we evaluate the performance of the proposed technique with some existing penalized methods including LASSO, elastic net, adaptive lasso and correlation based adaptive lasso. In order to cover the effects of outliers in various situations, we have taken the percentages of outliers as 0%, 10%, 20% and 30% from different distributions. The performances are evaluated in respect to their median prediction error, variable selection and bootstrap estimators.

The rest of the paper is organized as follows. Section 2, provides the background by briefly reviewing the residual function and weight function. We introduce the proposed robust penalized regression estimator in section 3. In section 4, we described the selection of the tuning parameters. Section 5 presents the results of the simulation studies. In section 6, we illustrate the performance of the proposed robust method on real data examples. Finally, section 7 offers some concluding remarks.

## 2 Background

Let *y*_1_, *y*_2_, …, *y*_*n*_ be an i.i.d random sample from a distribution *G*, having the density *g*, is modeled by the parametric functions *Ψ*_*θ*_ = {*F*_*θ*_: *θ* ∈ Θ ⊂ *R*^*d*^; *d* ≥ 1}. The maximum likelihood estimator(MLE) of *θ* is obtained by maximizing the likelihood $L\left(\theta \right)=\prod_{i=1}^{n}g_{\theta }\left(y_{i}\right)$. To obtain the MLE of *θ*, we solve the equation

$$\begin{array}{c} \frac{\partial }{\partial \theta }log_{e}L\left(\theta \right)=\sum_{i=1}^{n}u_{\theta }\left(y_{i}\right) \end{array}$$ (1)

In the present paper, we consider the solution to the weighted likelihood equation given by

$$\begin{array}{c} \sum_{i=1}^{n}w_{\theta }\left(y_{i}\right)u_{\theta }\left(y_{i}\right) \end{array}$$ (2)

The weights *w*_*θ*_(*y*_*i*_) will be constrained to lies between 0 and 1. This approach is motivated by the aim of generating estimators that are simultaneously robust and asymptotically fully efficient [23]. This technique borders on the idea of minimum disparity estimation proposed by [26] and [27].

[24] and [25] are considered the same approach that provide a quantification of the magnitude and sign of Pearson residuals, and are generally linked to a residual adjustment function employed in minimum disparity estimation. We consider the [23] weighting scheme which is downweighting of discrepant observations, where the strength of the downweighting increases steadily with the degree of discrepancy, or extreme outliers obtain weights close to 1.

In the following subsections, we briefly describe the residual function and weight function.

### 2.1 The residual function

Let *I*(.) denote an indicator function. We define *F*_*n*_ and *S*_*n*_ as $F_{n}\left(Y\right)=\frac{1}{n}\sum_{i=1}^{n}I\left(Y\leq y_{i}\right)$, $S_{n}\left(Y\right)=\frac{1}{n}\sum_{i=1}^{n}I\left(Y\geq y_{n}\right)$, these represent the empirical distribution function and survival function of the data. Let *F*_*θ*_(*Y*) = *P*(*Y* ≤ *y*) and *S*_*θ*_(*Y*) = *P*(*Y* ≥ *y*) be corresponding theoretical quantities. Now, the residual function *τ*_*n*_(*y*_*i*_) proposed by [23] is given as:

$$\begin{array}{c} \tau_{n}\left(y_{i}\right)=\{\;\begin{array}{c} \frac{F_{n}\left(y_{i}\right)}{F_{\theta }\left(y_{i}\right)}-1,\;if\;0<F_{\theta }\left(y_{i}\right)\leq q\\ 0,\;if\;q<F_{\theta }\left(y_{i}\right)<1-q\\ \frac{S_{n}\left(y_{i}\right)}{S_{\theta }\left(y_{i}\right)}-1,\;if\;1-q\leq F_{\theta }\left(y_{i}\right)<1 \end{array} \end{array}$$

Where *q* to choose is a suitable fraction *q* ≤ 0.5. This tuning parameter will determine the proportion of observations on either tail that will be subjected to possible downweighting. According to the above equation, it is clear that the consideration of the distribution function in the left tail and the survival function in the right tail helps to highlight the mismatch of the data and the model in the respective tails. Therefore, treat it as a case that requires downweighting.

### 2.2 The weight function

Now, we have our residual function, the next objective is to construct suitable weight function. The weight function should have the following properties:

(a) 0 ≤ *w*(*τ*_*n*_(*y*)) ≤ 1, *w*(0) = 1 (b) *w*(−1) is small, preferably close to 1.

[23] define residual adjustment function for weight as follows:

$$\begin{array}{c} H\left(y\right)=e^{-\alpha y^{2}}, \end{array}$$

Where *α* is a positive constant. There may be different forms for the downweighting structure represented by the function H(.). The role of this function has to be extensively studied in [24] and [25]. In this paper, we have used the above defined function.

Now, the weight function according to [23], can be defined as:

$$\begin{array}{c} w\left(\tau \right)=\{\begin{array}{c} 1,\;if\;q<F_{\theta }\left(y_{i}\right)<1-q\\ H\left(\frac{F_{n}\left(y\right)}{F_{\theta }\left(y\right)}-1\right),\;if\;F_{\theta }\left(y\right)\leq q\\ H\left(\frac{S_{n}\left(y\right)}{S_{\theta }\left(y\right)}-1\right),\;if\;F_{\theta }\left(y\right)\geq 1-q \end{array} \end{array}$$ (3)

## 3 Proposed Robust Adaptive Lasso: Penalizing the negative weighted log-likelihood

Consider the linear regression model

$$\begin{array}{c} y=X\beta +\varepsilon \end{array}$$ (4)

Where *y*_*n*×1_ is the response vector, *X*_*n*×*p*_ be the predictor matrix, *β* = (*β*_1_, …, *β*_*p*_)^*T*^ is the co-efficient vector, and *ε* = (*ε*_1_, …, *ε*_*n*_)^*T*^ is a vector of i.i.d random variables.

We consider the following regularization problem

$$\begin{array}{c} min_{\beta \in R^{p}}\{-n^{-1}\sum_{i=1}^{n}w\left(\tau \right)l_{i}\left(g\left(x_{i}^{T}\beta \right)\right)+\lambda_{n}\sum_{j=1}^{p}p_{\lambda_{n}}\left(\right|\beta_{j}\left|)\right\} \end{array}$$ (5)

where *w*(*τ*) is the weight function in Eq (3), *l*_*i*_(.) is the conditional log-likelihood, and *p*_λ_(.) is a non-negative penalty function on [0, ∞] with a regularization parameter λ_*n*_ ≥ 0.

The use of weight function in Eq (3) is overcome by the difficulty of heavy tails of the error distribution. It is non-negative, bounded above 1 and twice differentiable with respect to *τ*. The loss function for many models is convex, if the conditional distribution like *l*_*i*_(.), is from a class of the exponential family ([28]). The penalty function we will use is

$$\begin{array}{c} p_{\lambda_{n}}\left(.\right)=\lambda_{n}\sum_{j=1}^{p}\frac{|\beta_{j}|}{|{\hat{\beta }}_{init,j}|} \end{array}$$

where ${\hat{\beta }}_{init}$ is an initial estimator. In the present paper we get this from ridge regression estimator when *p* ≫ *n*, and in usual case (*n* > *p*) we used robust tukey bisquare M-estimator as initial estimator.

### 3.1 Theoretical properties

Consider the constant weight vector *w*, the penalized weighted log-likelihood function based on n samples is,

$$\begin{array}{c} Q_{n}\left(\beta \right)=W_{n}\left(\beta \right)-n\lambda_{n}\sum_{j=1}^{p}\frac{|\beta_{j}|}{|\beta_{init}|} \end{array}$$ (6)

Where *W*_*n*_(*β*) represent the weighted likelihood function. We write the true parameter coefficient vector as $\beta_{0}={\left(\beta_{10}^{T},\beta_{20}^{T}\right)}^{T}$, where *β*_10_ consists of all ‘s’ non-zero components and *β*_20_ consists of the remaining ‘s-1’ zero components. We write the corresponding maximizer of Eq (6) as $\hat{\beta_{n}}={\left({\hat{\beta }}_{1n}^{T},{\hat{\beta }}_{2n}^{T}\right)}^{T}$.

The Fisher Information matrix is

$$\begin{array}{c} I\left(\beta_{0}\right)=W.E\left\{\left[\frac{\partial l(\beta_{0})}{\partial \beta_{0}}\right]{\left[\frac{\partial l(\beta_{0})}{\partial \beta_{0}}\right]}^{T}\right\} \end{array}$$ (7)

Where *W* = *w*.*w*^*T*^ is (1 × *n*) positive definite matrix. Let *I*_1_(*β*_10_) = *I*_11_(*β*_10_, 0), where *I*_11_(*β*_10_, 0) is the leading (*s* × *s*) submatrix of *I*(*β*_0_) with *β*_20_ = 0. We also assume that if $\sqrt{n}\lambda_{n}=O_{p}\left(1\right)$, then the adaptive Lasso type estimator satisfies $\left|\right|\hat{\beta_{n}}-\beta_{0}\left|\right|=O_{p}\left(n^{-\frac{1}{n}}\right)$. It shows that $\hat{\beta_{n}}$ is root n-consistent if λ_*n*_ ↦ 0 ([7]). Next we show that, when λ_*n*_ is chosen properly the proposed Robust estimator has the oracle property under the same regularity conditions of [7].

**Theorem**: *Assume that* $\sqrt{n}\lambda_{n}\;\mapsto \;0$ *and nλ*_*n*_ ↦ ∞. *Then under the some regularity conditions*, *with probability tending to 1*, *the root n-consistent adaptive Lasso Robust estimator* $\hat{\beta_{n}}$ *must satisfy the following properties*.

(i) *Sparsity*; $\hat{\beta_{2n}}=0$.

(ii) *Asymptotic normality*; $\sqrt{n}\left(\hat{\beta_{1n}}-\beta_{10}\right)\;\mapsto \;N\left(0,W.I_{1}^{-1}\left(\beta_{10}\right)\right)$ *in distribution as n* ↦ ∞.

The proofs of theorem follows the same steps of [7], under same regulaty conditions.

## 4 Selection of the tuning parameters

We now discuss an important issue of the tuning parameters selection regarding the construction of weight functions and in the different regularization methods. For the weight functions, increasing the value of the parameter “*α*” leads to greater downweighting. To make balance between the degree of robustness and efficiency, we need an extensive numerical studies. In the present simulation study we keep it in the range of (0.05, 0.005). This appears to be a difficult problem, but solving this issue remains among our plan for future work.

On the other hand, in the different regularization methods described in this paper, we need to choose an optimal tuning parameters $\lambda_{n}^{\prime }s$. Intuitively, an optimals of these tuning parameters be chosen through a variety of tools such as cross validation (CV), generalized CV and Bayesian information criterion (BIC) approach.

There are well-established methods for choosing such parameters [29]. [17] and [30] used BIC-type criterion, and [3] using 10-fold CV technique and proved that the resulting estimator with such type of tuning parameters satisfies the good prediction accuracy and model selection. Following this idea, we apply 10-fold CV procedure for selection of tuning parameters.

## 5 Simulation studies

In this section, we introduced some numerical examples to illustrate the performance of the Robust Adaptive Lasso method described in section (3) with other methods. We set two scenarios in every example in terms of pairwise correlation between predictors, i.e, *r* = 0.5 and 0.85. Besides this, four levels of contamination were considered in error distribution (*δ* = 0%, 10%, 20% and 30%) from two types of contamination, that is, scale and location contamination.

The following four performance measures were calculated:

(a) Prediction Error- computed on the test data set.

(b) Bootstrap Standard Error-by using the bootstrapp with B = 500 resamplings on the 1000 mean squared errors (in percentage).

(c) The average number of “0” estimated coefficients correctly, denoted by “*C*”.

(d) The average number of “0” estimated coefficients incorrectly, denoted by “*I*”.

We simulated 1000 data sets for each example from the linear regression model

$$\begin{array}{c} y_{i}=x_{i}^{T}\beta +\varepsilon_{i} \end{array}$$

The predictors, *X*_*i*_, …, *X*_*p*_ were generated from the multivariate normal distribution, *N*(0, *Σ*) with *Σ* = (*ρ*_*jk*_)_*p*×*p*_ and *ρ*_*jk*_ = *r*^|*j*−*k*|^. For the distribution of the noise, *ε*_*i*_, we considered *N*(0, 1) with different levels of contamination from symmetric and asymmetric distribution.

**Scenario-I**: We let *β* = (3, 1.5, 0, 0, 2, 0, 0, 0). We consider two cases for sample size, i.e, n = 50, and 100. Next, we took a scenario where an error distribution to be (1 − *δ*)*N*(0, 1) + *δN*(−10, 1) which is contaminated by *N*(−10, 1) with different levels of percentages, *δ*.

**Scenario-II**: This scenario is the same as first, except that the error distribution to be (1 − *δ*)*N*(0, 1) + *δN*(0, 25).

**Scenario-III**: In this case we consider the sample size n = 120 and covariates dimension *p* = 400. We set the coefficient vector *β* in which first 15 components are non-zero and the remaining are zero. Given is, *β* = {(3, …, 3)_5_, (1.5, …, 1.5)_5_, (2, …, 2)_5_, (0, …, 0)_385_}.

**Scenario-IV**: This scenario is same as scenario-I, except that the error distribution is *exponential*(1) contaminated by an *exponential*(1/5).

**Scenario-V**: In this case, we demonstrate the *p* ≫ *n* situation, when errors have non-normal distributions. We use *n* = 120 observations and *p* = 400 predictors. We sample *ϵ*_*i*_ from exponential distribution, i.e, (1 − *δ*)*exp*(1) + *δexp*(1/5).

We fixed the true regression coefficients vector as, *β* = {(3, …, 3)_5_, (1.5, …, 1.5)_5_, (2, …, 2)_5_, (0, …, 0)_385_}

Table 1 summarized the simulation results in scenario-I, for the different levels of contamination, different sample sizes, and low and high pairwise correlation between predictors. To measure the quality of the proposed technique, median test error, bootstrap standard error and predictor selection performance were computed under each condition.

**Table 1:** Simulation results for the location level of contamination in the model(1 − *δ*)*N*(0, 1) + *δN*(−10, 1).

(*n*, *p*)	*δ*	Method		r = 0.5			r = 0.85	
			Med.PMSE	C	I	Med.PMSE	C	I
(50,8)	0%	Lasso	1.448(1.58)	4.384	0	1.361(1.76)	5	0
		E.net	1.479(1.82)	2.388	0	1.387(1.71)	2.242	0
		Alasso	1.335(1.83)	4.837	0	1.405(2.03)	3	1
		CBPR	1.202(1.46)	3.593	0	1.311(1.68)	3.512	0
		*RAL*	1.254(1.33)	4.713	0	1.430(1.72)	2.62	0
	10%	Lasso	1.187(1.53)	0.422	0	1.131(1.31)	4	0
		E.net	1.198(1.60)	1.946	0	1.161(1.45)	1.532	0
		Alasso	1.124(1.89)	4.568	0	1.208(2.05)	3	0.094
		CBPR	1.100(1.30)	4.798	0	1.076(1.19)	4	0
		*RAL*	1.001(1.02)	3.913	0	1.029(1.24)	4.837	0
	20%	Lasso	0.969(1.34)	3.084	0.02	0.930(1.16)	5	1
		E.net	1.011(1.39)	2.189	0	0.939(1.20)	1	0
		Alasso	0.983(2.62)	3.993	0	1.082(2.54)	2.386	1.756
		CBPR	0.900(1.12)	3.268	0	0.920(1.11)	2.704	0
		*RAL*	0.850(0.92)	4.735	0	0.823(0.89)	4.910	0
	30%	Lasso	0.801(0.95)	3.004	0	0.779(0.98)	3.296	0
		E.net	0.837(1.13)	2.358	0	0.807(0.99)	1.935	0
		Alasso	0.865(3.65)	3	0	1.001(3.42)	3.998	0.014
		CBPR	0.753(0.96)	4.006	0	0.802(0.98)	4.113	0
		*RAL*	0.704(0.80)	4.442	0	0.707(0.81)	5	0
(100,8)	0%	Lasso	1.187(0.83)	3	0	1.169(0.79)	4.621	0
		E.net	1.215(0.85)	1.576	0	1.179(0.77)	2.231	0
		Alasso	1.140(0.87)	4.897	0	1.162(0.82)	4.003	0
		CBPR	1.210(0.75)	3.457	0	1.139(0.78)	5	0
		*RAL*	1.100(0.70)	4.365	0	1.121(0.73)	4.159	0
	10%	Lasso	0.964(0.69)	3	0	0.956(0.64)	4	0
		E.net	0.981(0.67)	3.706	0	0.968(0.70)	3.714	0
		Alasso	0.934(0.62)	3.232	0	0.937(0.71)	4.5	0.002
		CBPR	0.932(0.61)	4	0	0.938(0.62)	3.602	0
		*RAL*	0.895(0.60)	4.578	0	0.910(0.51)	4.330	0
	20%	Lasso	0.805(0.61)	3.065	0	0.791(0.56)	4	0
		E.net	0.812(0.54)	2.498	0	0.793(0.53)	2.107	0
		Alasso	0.773(0.60)	4.597	0	0.809(0.90)	3.084	0
		CBPR	0.784(0.55)	3.616	0	0.775(0.50)	3.272	0
		*RAL*	0.758(0.54)	5	0	0.745(0.50)	4.530	0
	30%	Lasso	0.678(0.45)	4	0	0.667(0.47)	3	0
		E.net	0.697(0.54)	1.296	0	0.684(0.48)	3.98	0
		Alasso	0.666(0.84)	4.483	0.01	0.740(1.13)	4.15	0.012
		CBPR	0.664(0.46)	5	0	0.672(0.44)	4	0.09
		*RAL*	0.649(0.40)	4.805	0	0.630(0.39)	5	0

### Effects of sample size

We observe that, across sample sizes, the median test error and bootstrap standard error decreased with the increasing sample size from 50 to 100 in both cases of low and high correlation among predictors. This pattern of decreasing holds for all regularization methods, but the decrease in the proposed Robust adaptive Lasso is more than other competitors. On the other hand, in terms of variable selection with the increase of sample size, the ratio of incorrectly “0” selection of coefficients, denoted by “I”, decreases significantly. Table 1 also shows that for, n = 100, the correct selection, denoted by “C”, is improved as compared for n = 50, but the proposed Robust adaptive Lasso methods, defeated all other methods, in both cases, i.e, in “C” and “I” and performs just like oracle estimator (i.e, C = 5 and I = 0).

### Effects of level of contamination

Under ideal condition (unit normal error distribution, *δ* = 0%), the results in Table 1 indicate that the “CBPR” and proposed method provides good results in terms of both prediction and variable selection, particularly in r = 0.85, the predictor selection performance is the nearest to oracle estimator, but in, n = 100, case, the test error of the proposed method is lower than all other methods.

For the 10% data contamination condition, the proposed Robust adaptive Lasso shows strong performance in terms of both prediction accuracy and variable selection, but in only one cell, i.e, for n = 50 and *δ* = 10%, the variable selection performance is not better than CBPR.

From Table 1, it is clear that the proposed method shows superior performance under 20% and 30% contamination conditions for both cases of sample sizes and correlations among predictors, respectively. In this extreme location contamination, the proposed Robust method has performed just like an oracle estimator and also has minimum bootstrap standard errors with excellent predictor selection performance. Hence, the overall performance of proposed the Robust adaptive Lasso method is better than other with the increase of location contamination.

### Effects of multicollinearity

In given scenario-I, we also consider two cases for correlation between predictors, i.e, r = 0.5 and 0.85. In both situations, the Robust adaptive Lasso method outperforms all other methods, except CBPR which has a little better result in case of high correlation and clean data (*δ* = 0%), particularly, in variable selection. But, the levels of contamination increases in both low and high collinearity. The proposed method findings become better and better. From Table 1, we can also see that the [8] gives very poor results in high correlation between predictors, especially, in variable selection.

Simulation results for scenario-II are summarized in Table 2, in which we consider scale level of contamination scheme where *N*(0, 1) model is contaminated by *N*(0, 25). The details are discussed as below.

**Table 2:** Simulation results for the scale level of contamination in the model(1 − *δ*)*N*(0, 1) + *δN*(0, 25).

(*n*, *p*)	*δ*	Method		r = 0.5			r = 0.85	
			Med.PMSE	C	I	Med.PMSE	C	I
(50,8)	0%	Lasso	1.390(1.70)	3.411	0	1.372(1.62)	3.022	0
		E.net	1.486(1.78)	1.990	0	1.390(1.72)	2.683	0
		Alasso	1.357(1.91)	4.851	0	1.431(1.97)	4.543	0.07
		CBPR	1.335(1.65)	3.751	0	1.325(1.37)	3.337	0
		*RAL*	1.262(1.35)	4.658	0	1.262(1.33)	4.586	0
	10%	Lasso	10.246(13.64)	2.103	0	9.521(10.76)	3.938	0
		E.net	9.986(12.59)	2.126	0	9.402(10.27)	2.587	0
		Alasso	10.445(15.52)	2.839	0.447	10.037(11.99)	2	0.747
		CBPR	10.262(12.79)	3.497	0	9.619(10.40)	2.940	0.424
		*RAL*	9.215(11.76)	3.451	0	8.720(9.32)	4.07	0
	20%	Lasso	34.591(41.62)	2.191	0.382	32.384(35.41)	3.603	0
		E.net	34.014(40.81)	0.976	0.776	32.458(38.22)	1.934	0.92
		Alasso	37.373(52.49)	3.515	0.64	35.498(46.89)	0	1
		CBPR	37.649(48.59)	1	0	31.654(33.38)	1.730	1.645
		*RAL*	32.768(41.57)	3.972	0	30.902(33.21)	4.369	0.043
	30%	Lasso	73.419(82.02)	1.909	1	69.504(76.62)	3.213	0.921
		E.net	73.889(90.57)	0.958	0	68.915(91.13)	0.870	1.793
		Alasso	81.505(97.31)	4	1.749	76.578(96.12)	1.948	2.01
		CBPR	74.194(98.32)	3.370	0.034	69.384(80.65)	3.265	2
		*RAL*	72.284(93.72)	4.639	0	67.299(82.93)	4.037	0.745
(100,8)	0%	Lasso	1.194(0.78)	3.07	0	1.162(0.79)	4.007	0
		E.net	1.190(0.87)	2.488	0	1.199(0.83)	1	0
		Alasso	1.150(0.78)	4.524	0	1.159(0.84)	4.012	0
		CBPR	1.159(0.80)	3.752	0	1.161(0.80)	3.157	0
		*RAL*	1.113(0.70)	4.866	0	1.102(0.68)	5	0
	10%	Lasso	8.487(5.71)	1.106	0	8.165(5.31)	4.795	0.02
		E.net	8.547(6.11)	2.318	0	8.155(5.47)	3.340	0
		Alasso	8.125(5.75)	3.759	0	8.431(5.56)	2.992	0
		CBPR	8.294(5.65)	3.456	0	8.108(5.38)	3.335	0
		*RAL*	8.012(5.45)	4.307	0	7.491(4.93)	4.267	0
	20%	Lasso	30.023(19.73)	1.086	0	29.266(19.08)	3.811	0.809
		E.net	29.779(19.84)	2.259	0	29.768(18.34)	0.960	0
		Alasso	30.922(21.29)	2	0.805	29.275(19.77)	2.99	1.982
		CBPR	29.539(20.40)	2.82	0	29.056(19.04)	4.392	0.948
		*RAL*	29.322(18.19)	4.72	0	28.592(18.94)	4.572	0.114
	30%	Lasso	66.129(44.12)	2.474	0.959	62.991(42.60)	1.749	1.808
		E.net	64.387(41.11)	2.014	0	63.316(39.76)	3.268	0.230
		Alasso	67.047(44.33)	3.011	2	65.929(43.11)	2.847	0.639
		CBPR	65.303(43.02)	3.685	0	63.563(39.06)	3.506	0.715
		*RAL*	63.560(44.96)	4.659	0	63.039(42.10)	4.078	0.039

### Effects of sample size

From the findings given in Table 2, it is evident that the prediction errors and bootstrap standard errors of all regularization approaches blows up in the presence of scale contamination. The results shows that when sample size doubled from 50 to 100, the test errors and as well as bootstrap standard errors significantly decreases, for fixed contamination and correlation among predictors. Among these five methods, the reduction in the proposed Robust method is maximum. We can also observe that, the variable selection performance is also improved when the sample size increases, particularly for the proposed method, in terms of both aspects “C” and “I”. Generally speaking, the usual positive effect of sample size is on prediction accuracy and standard error.

### Effects of level of contamination

It is clear form Table 2, that the proposed Robust regularization technique provides good results in terms of model error in all cases of contamination. The variable selection performance of Lasso and [8] are extremely poor when the contamination rate increases from% to 30%. The findings also indicate that the proposed method remains almost consistent in variable selection under low and high contamination, as compared to other competitors.

### Effects of multicollinearity

From Table 2, it can be shown that in both cases of correlation, i.e, r = 0.5 and r = 0.85, the proposed Robust method is the best one overall. But it is very interesting to see that the Robust RAL method in terms of test errors and bootstrap standard errors is relatively better when the predictors are highly correlated, but in predictor selection in high correlation and extreme contamination aspect (i.e, 20% and 30%) is worse than low correlation condition among predictors. For example, in case of fixed (*n*, *p*) = (50, 8) and *δ* = 30%, the proposed Robust method estimated 4.639 predictors on the average, in “C” and in aspect of “I” is 0, when *r* = 0.5. But on the other hand, in *r* = 0.85, the situation is 4.037 in aspect of “C” and 0.745 in “I”, respectively.

In scenario-III, the predictors dimension is larger than sample observations, i.e, *p* = 400 and *n* = 120, but the dimension of the true model is fixed to be 15. The detailed results are depicted in Table 3. To compare the performance of the Robust Adaptive Lasso estimator, we did not report the results of the [8], because in case of, *p* ≫ *n*, it is nontrivial to calculate reliable initial estimates (i.e, OLS estimators) for weights used in it. From Table 3, the results confirm robustness of the proposed method with increasing the level of contamination, and hence, the prediction error and bootstrap standard errors decrease slowly when *δ* increases towards 30%.

**Table 3:** Simulation results for the location level of contamination in the model(1 − *δ*)*N*(0, 1) + *δN*(−10, 1) in high-dimensional data set.

*δ*	Method		r = 0.5			r = 0.85	
		Med.PMSE	C	I	Med.PMSE	C	I
0%	Lasso	5.413(27.8)	379.001	1.003	2.017(2.00)	384.3	3.17
	E.net	6.639(23.2)	379.003	0	1.957(1.98)	383.043	0.14
	CBPR	2.410(2.20)	376.620	0	2.257(1.93)	366.941	0
	*RAL*	2.054(1.50)	370.820	0	2.052(2.00)	373.156	0
10%	Lasso	8.691(27.20)	372.002	2.005	1.640(1.66)	376.106	0
	E.net	4.942(22.2)	379.995	0	1.576(1.63)	373.850	0
	CBPR	1.687(1.50)	381.009	0	1.674(1.51)	363.644	0
	*RAL*	1.894(1.01)	369.526	0	1.400(1.20)	379.245	0
20%	Lasso	4.896(27.6)	379.002	2.001	1.369(1.49)	376.624	0
	E.net	5.769(22.5)	379.001	0	1.357(1.35)	373.523	0
	CBPR	1.975(1.80)	381.012	0	1.213(1.03)	375.590	0
	*RAL*	1.678(1.20)	370.550	0	1.181(1.04)	377.505	0
30%	Lasso	3.140(35.07)	364.230	0.005	1.178(1.23)	369.374	0
	E.net	4.361(32.05)	357.672	0	1.150(1.33)	374.236	0
	CBPR	2.273(1.63)	368.892	0	1.103(0.96)	369.537	0
	*RAL*	1.297(1.66)	370.747	0	0.152(1.05)	381.078	0

In case of variable selection, from Table 3, it can be seen that the predictor selection results of the other methods have a little more advantage than the proposed method in the aspect of “C”, especially for low level of contamination and moderate correlation, i.e, *r* = 0.5, but in extreme contamination condition, i.e, (*δ* = 30%) the performance of Robust proposed method becomes better than others. On the other hand, in high correlation set up among predictors, the proposed technique gives satisfactory results in both aspects of “C” and “I”, except for clean data, i.e, (*δ* = 0%).

In scenario-IV, we consider the non-normal error distribution. The Table 4, presents the prediction error, bootstrap standard error and predictor selection performance when the error has heavy tailed an exponential(1) distribution, contaminated by an exponential (1/5) distribution. From Table 4, it may be seen that the contamination proportion tends to the level of 30%, the prediction error of the proposed penalized regression method is stable and does not increases as compared to others. In terms of predictor selection, the proposed robust method remains better for higher level of contamination too, for fixed sample size and correlation ammong predictors. Additionally, for the small sample size Table 4 reported that the test errors for the different procedures are grater than in case of large sample size (i.e, n = 100), for all fixed cases of conatmination and correlation.

**Table 4:** Simulation results are based on 1000 replications and *δ* is the level of contamination in the model (1 − *δ*)*exp*(1) + *δexp*(0.2).

(*n*, *p*)	*δ*	Method		r = 0.5			r = 0.85	
			Med.PMSE	C	I	Med.PMSE	C	I
(50,8)	0%	Lasso	1.354(2.73)	3.325	0	1.333(2.43)	2.660	1
		E.net	1.336(2.41)	1.669	0	1.356(2.48)	1.795	0
		Alasso	1.282(4.11)	3.648	0	1.389(3.22)	4.382	0
		CBPR	1.375(2.60)	3.204	0.007	1.321(2.33)	3.179	0
		*RAL*	1.192(2.21)	4.639	0	1.227(2.09)	4.575	0
	10%	Lasso	1.455(2.48)	1.554	0	1.414(2.10)	3.617	0
		E.net	1.494(2.60)	0.540	0	1.394(2.22)	1.832	1
		Alasso	1.385(3.13)	4.064	0	1.610(3.19)	3	0
		CBPR	1.372(2.24)	4.590	0	1.375(2.34)	2.06	0
		*RAL*	1.253(2.06)	4.892	0	1.243(2.00)	4.668	0
	20%	Lasso	2.226(3.68)	2.311	0	2.117(3.16)	2.323	1
		E.net	2.305(4.05)	2.373	0	2.256(3.35)	2.829	0
		Alasso	2.382(4.85)	4.249	0.021	2.480(4.58)	4.163	1
		CBPR	2.134(3.36)	3.472	0.002	2.157(3.02)	3.619	0
		*RAL*	1.953(2.89)	4.949	0	2.016(2.81)	4.888	0.001
	30%	Lasso	3.800(6.33)	2.393	0	3.567(5.71)	4.091	0.17
		E.net	3.810(6.51)	1.522	0	3.508(6.24)	3.261	0
		Alasso	3.941(8.40)	3.930	0.052	3.908(6.68)	3.599	0.233
		CBPR	3.493(5.85)	3.719	0	3.527(6.40)	3.843	0
		*RAL*	2.468(5.42)	4.221	0	2.469(5.32)	4.489	0.04
(100,8)	0%	Lasso	1.135(1.27)	3.198	0	1.154(0.81)	3	0
		E.net	1.133(1.28)	1.421	0	1.185(0.80)	1.62	0
		Alasso	1.081(1.33)	4.859	0	1.158(0.78)	4.99	0
		CBPR	1.103(1.29)	3.069	0	1.111(0.77)	4.73	0
		*RAL*	1.173(1.44)	3.473	0	1.106(0.74)	5	0
	10%	Lasso	1.205(1.19)	3.117	0	1.222(0.90)	3	0
		E.net	1.235(1.25)	0.388	0	1.235(0.92)	1.361	0
		Alasso	1.176(1.14)	4.189	0	1.232(0.88)	2.943	1
		CBPR	1.166(1.30)	3.683	0	1.220(0.85)	4	0
		*RAL*	1.131(1.17)	4.228	0	1.181(0.81)	4.430	0
	20%	Lasso	1.925(1.98)	2.004	0	1.863(1.64)	3.329	0
		E.net	1.935(1.88)	1.547	0	1.876(1.72)	3.564	0
		Alasso	1.860(1.85)	3.007	0	1.898(1.82)	3.519	0
		CBPR	1.786(1.69)	4.166	0	1.868(1.60)	2.015	0
		*RAL*	1.062(1.68)	4.301	0	1.754(1.55)	4.865	0
	30%	Lasso	3.130(3.18)	2.418	0.031	3.091(3.09)	3.001	0
		E.net	3.203(3.64)	1.883	0	3.090(3.03)	2.554	0
		Alasso	3.148(3.56)	3.370	0.926	3.240(3.52)	1	0.001
		CBPR	3.043(3.26)	4.392	0.012	3.075(3.21)	2.957	0
		*RAL*	2.960(3.00)	4.768	0	2.939(2.98)	4.614	0

Our simulation results for scenario-V are depicted in Table 5. For the exponential error distribution, the robust adaptive Lasso exhibits good prediction performance in both low and high correlations among predictors when contamination tends to 30%. In terms of variable selection, we observe that the performance of the proposed method tends to dominate the other penalized regression procedures when the level of contamination increases towards 30%. These findigs suggest that the proposed robust procedure, by utilizing weights proposed by [23], is effective when the tails got heavier.

**Table 5:** Simulation results for the location level of contamination in the model(1 − *δ*)*exp*(1) + *δexp*(0.2) in high-dimensional data set.

*δ*	Method		r = 0.5			r = 0.85	
		Med.PMSE	C	I	Med.PMSE	C	I
0%	Lasso	5.170(36.74)	354.096	2.143	2.057(2.12)	374.469	0
	E.net	6.704(33.29)	352.160	1	1.982(1.81)	375.198	0
	CBPR	2.684(3.97)	368.523	0	1.707(1.49)	376.233	0
	*RAL*	2.878(4.37)	367.174	0	1.677(1.55)	380.644	0
10%	Lasso	5.763(35.56)	363.011	0.034	2.160(2.26)	370.476	0.015
	E.net	7.306(37.42)	354.849	0	2.072(2.18)	373.299	0
	CBPR	3.919(5.57)	359.637	0	1.866(1.66)	372.595	0
	*RAL*	3.881(5.10)	367.295	0	1.892(1.58)	381.919	0
20%	Lasso	8.281(32.98)	364.791	0.122	3.300(3.63)	370.232	0
	E.net	9.923(37.64)	356.168	0	3.116(3.48)	375.916	0
	CBPR	3.682(5.37)	369.929	0	3.947(3.10)	366.586	0
	*RAL*	2.551(4.43)	370.475	0	2.813(2.95)	380.964	0
30%	Lasso	13.869(46.19)	361.1	0.233	5.369(6.33)	371.226	0
	E.net	15.110(44.32)	347.852	0	5.092(5.55)	375.691	0.108
	CBPR	5.566(8.13)	365.307	0	5.391(6.381)	363.874	0
	*RAL*	4.659(7.91)	377.308	0	4.263(4.92)	379.011	0

## 6 Real data application

### 6.1 Prostate cancer data

The data set for this subsection comes from a study by [31] and analyzed by [29] for estimation and variable selection. The data set consists of 97 observations and 9 variables. We used the response variable which is “lcavol” and the rest are explanatory variables. The proposed Robust penalized method was applied along with four other penalized approaches (i.e, given in Table 6). The first 30 observations were used as training data set and the rest as testing data to evaluate the prediction ability.

**Table 6:** Estimated coefficients and model error results, for various regularization procedures applied to the prostate data. The dashed entries correspond to predictors that are estimated “0”.

Variable	Lasso	Elastic net	Alasso	CBPR	*RAL*
Intercept	0.635	0.206	2.921	1.844	0.208
lweight	−0.282	…	−0.821	−0.425	…
age	0.027	0.008	…	0.029	…
lbph	…	…	…	…	…
svi	…	…	…	…	…
lcp	1.135	0.836	1.213	1.266	0.648
gleason	0.088	0.102	0.267	…	…
pgg45	…	…	…	…	…
lpsa	0.094	0.05	0.374	…	0.342
Test Error	1.997	1.132	3.112	2.413	0.807

**Fig 1:**
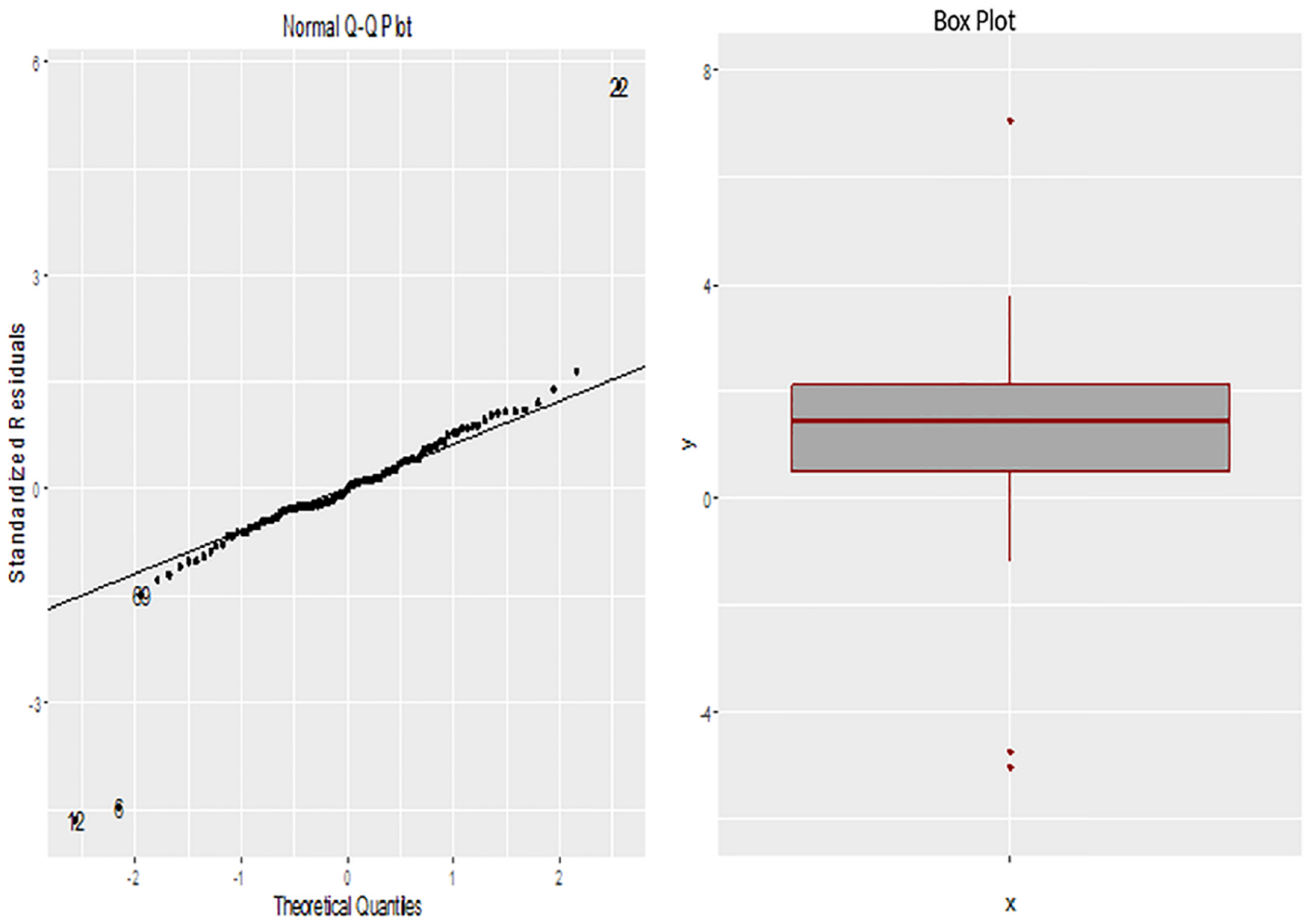
The Q-Q plot and boxplot of the response variable “lcavol”.

In Fig 1, the QQ-plot and boxplot shows that there are three distinct outliers in the response variable. A normal model would provide a nice fit to response variable if the outliers are deleted. We set our optimum tuning parameters values as *q* = 30, that is to determine the proportion of observations on either tail that will be subjected to downweighting, and “*α* = 2.202” for proposed approach. Beside this, the weights associated with these three identified outliers are, 0.0380, 0.0092 and 0.0359, respectively.

The columns of Table 6 present the different penalized approaches with the proposed robust method, their associated coefficient estimates and test errors. From the Table 6, we can see that the proposed method produces more sparse solution and selects two covariates, i.e, “lcp” and “lpsa”, corresponding to the smallest test error, i.e, 0.807.

### 6.2 Microarray data-riboflavin production by bacillus subtilis

Here we analyze the high-dimensional real data set (S2 File) about riboflavin production by bacillus subtilis which was analyzed by [32]. Here the continuous response variable Y which measures the logarithm of the production rate of riboflavin, p = 4088, is the number of covariates corresponding to the logarithms of expression levels of genes and n = 71 individuals of genetically homogeneous sample.

Our main objective here is to test whether our method can effectively select covariates with non-zero coefficients and estimate parameters simultaneously.

**Fig 2:**
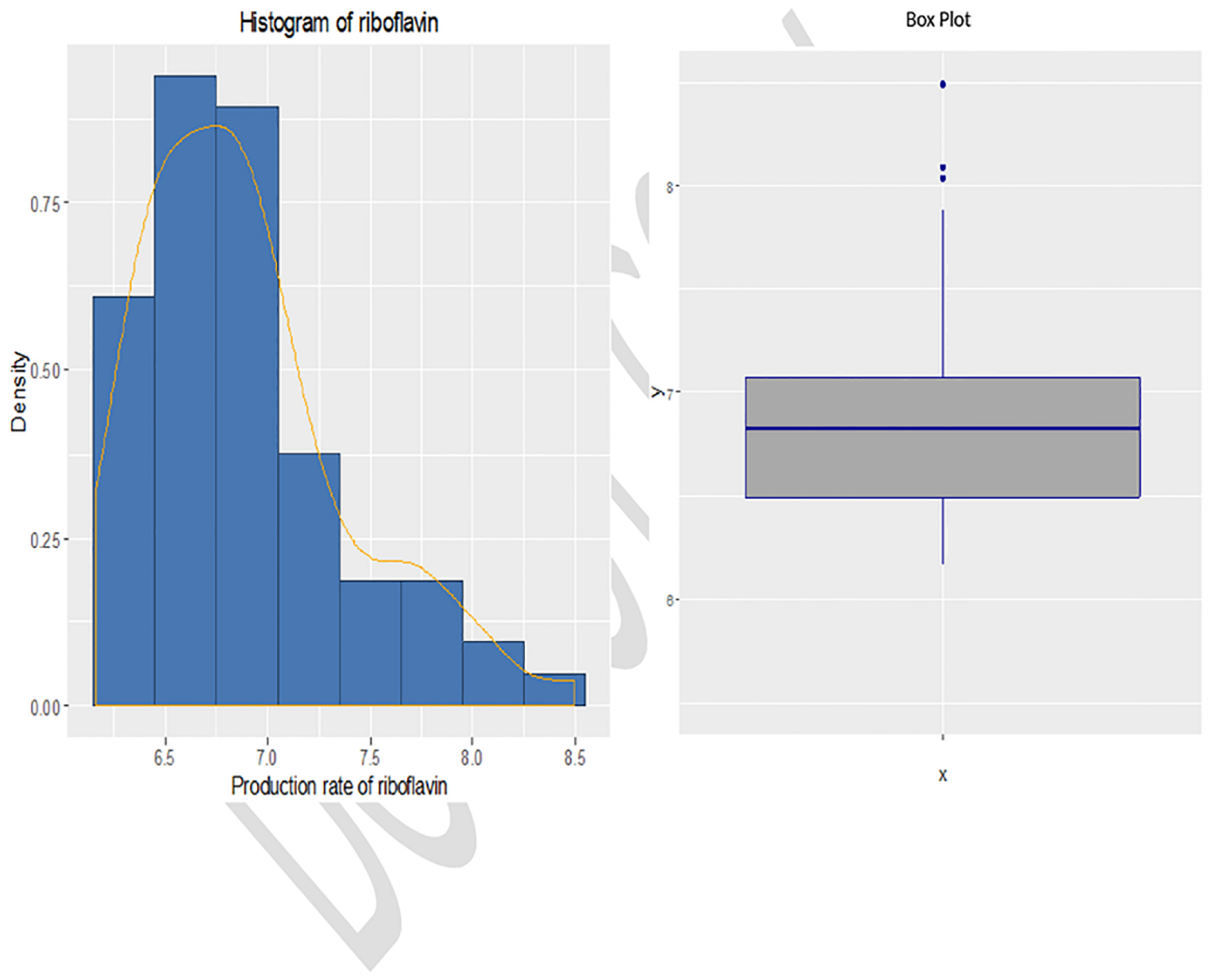
(a) Histogram and (b) Boxplot for production rate in riboflavin.

From Fig 2(a), it can be observed that the frequency distribution of the response variable is somehow positively skewed and the boxplot in Fig 2(b) clearly shows that there are some outlying observations present in the data. Since, the response variable is skewed, therefore, gamma distribution was fitted on it after applying K-S test (i.e, p-value>0.05). For the purpose of the proposed weights, we set our tuning parameters values as *q* = 0.5 and *α* = 0.312.

**Fig 3:**
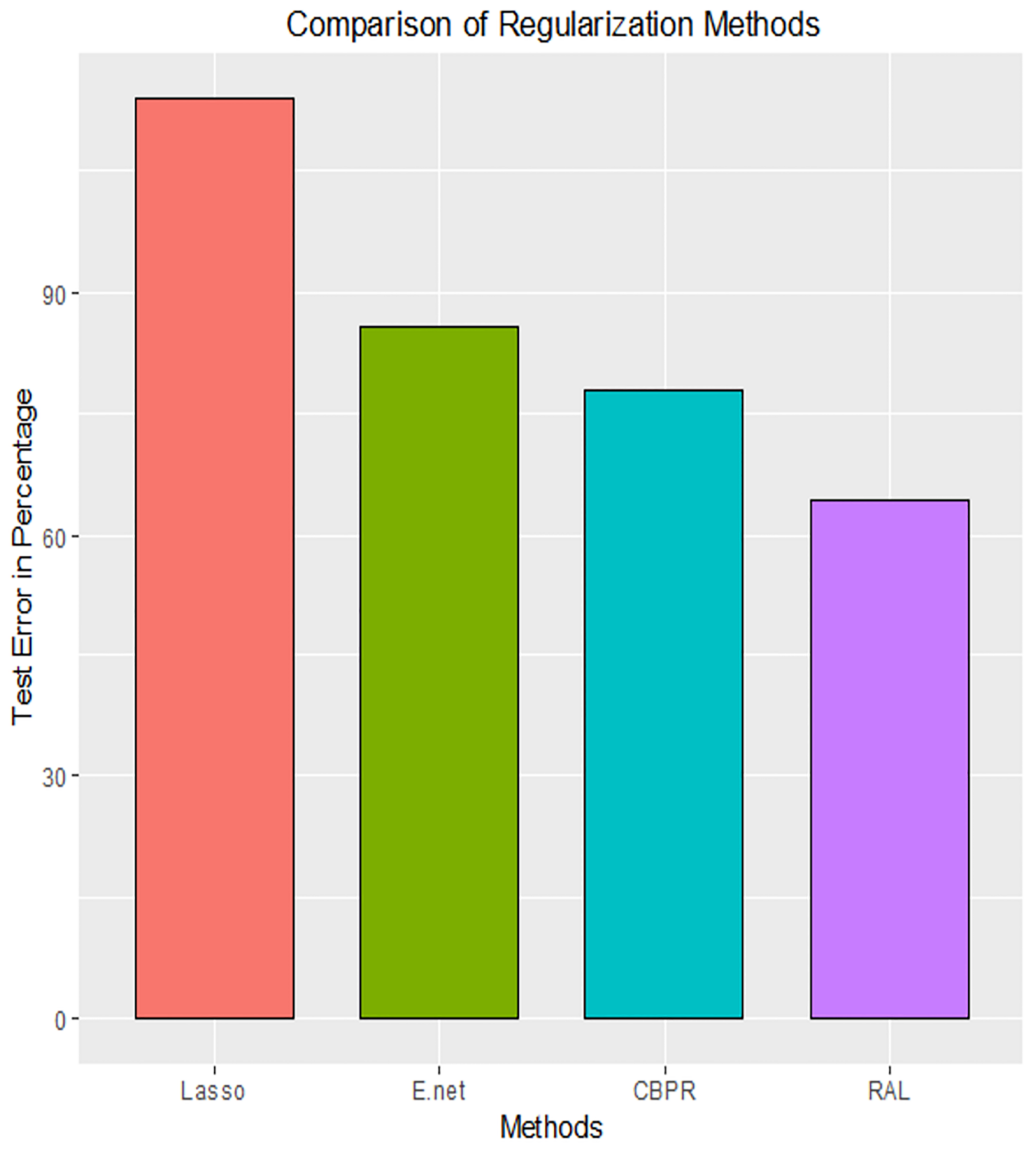
Comparison of percentage test error.

Percentage prediction/test errors were calculated for the five regularization regression techniques, and were compared with percentage test error of the ridge regression (i.e, 0.0829). The percentage errors were shown by a bar graph in Fig 3. It can be seen from Fig 3 that Lasso performs very poorly with percentage test error which is 113.915%, and which is 13.915% more than ridge regression. The prediction error of elastic net is 14.21%, (i.e, 85.79%) lower and CBPR is 22.08%, (i.e, 77.92%) lower than ridge penalized regression. Among these methods the maximum reduction in percentage test error is observed in the proposed penalized regression procedure with percentage test error is 64.29%, which is 35.71% lower than ridge method.

In terms of sparsity, the number of non-zero estimated predictors coefficients of Lasso, elastic net, CBPR and RAL are 16, 32, 42 and 35, respectively, out of the total number of 4088 predictors. Since, the proposed RAL method selects 35 covariates with minimum prediction error.

## 7 Conclusion

In this article we proposed a robust penalized regression model (RAL) using weighted log-likelihood with the adpative Lasso penalty function. We used the [23] proposed weight function to downweights the points that are large residuals outliers and improved the effectiveness of the proposed algorithm. Four penalization methods in addition to RAL, including Lasso, elastic net, adaptive lasso and CBPR were compared. The numerical simulations shows that for high percentages of contamination the RAL is more robust and outperforms the other penalization procedures interms of prediction accuracy, bootstrapped standard errors and variable selection.

We also illustrate the proposed method in an application to real data analysis. we consider the prostate cancer data set and a high-dimensional data set about riboflavin (Vatamin *B*_2_) production by bacillus subtilis. We have evaluated the performance of the different penalized procedures based on training/testing sample partition. The real data comparison in section 6, demonstrate that the proposed robust procedutre (RAL) improves over existing methods in both prediction and varaible selection. Thus, RAL is more robust procedure than other methods to outliers or influential observations.

## Supporting information

S1 File Data set1. (CSV)

S2 File Data set2. (CSV)
